# Impact of Seniors Centers on oral health-related quality of life of older adults

**DOI:** 10.11606/s1518-8787.2020054001648

**Published:** 2020-01-14

**Authors:** Fernanda W. Machado Luz, Alexandre Emídio Ribeiro Silva, Ana Paula Perroni, Marília L. Goettems, Noéli Boscato

**Affiliations:** I Universidade Federal de Pelotas. Faculdade de Odontologia. Programa de Pós-Graduação em Odontologia. Pelotas, RS, Brasil; II Universidade Federal de Pelotas. Programa de Pós-Graduação em Odontologia. Departamento de Odontologia Social e Preventiva. Pelotas, RS, Brasil; III Universidade Federal de Pelotas. Faculdade de Odontologia. Programa de Pós-Graduação em Odontologia. Pelotas, RS, Brasil; IV Universidade Federal de Pelotas. Programa de Pós-Graduação em Odontologia. Departamento de Odontologia Social e Preventiva. Pelotas, RS, Brasil; V Universidade Federal de Pelotas. Programa de Pós-Graduação em Odontologia. Departamento de Odontologia Restauradora. Pelotas, RS, Brasil

**Keywords:** Aged, Self-Help Groups, Oral Health, Quality of Life

## Abstract

**OBJECTIVE:**

This study evaluated the oral health-related quality of life (OHRQoL) of older adults participating or not in Seniors Centers (SC).

**METHODS:**

Two independent samples were compared: older adults who participate in SC (n = 124) and older adults who visited Primary Healthcare Centers (PHC) and do not participate in SC (n = 164). The data collected consisted of sociodemographic (sex, age, educational level, marital status, family income) and psychosocial characteristics—Sense of Coherence (SOC), anxiety and depression using HADS, happiness—, and oral clinical evaluation—use and need of dental prosthesis and decayed (D), missing (M), or filled (F) teeth. The resulting OHRQoL was evaluated using the Oral Health Impact Profile (OHIP-14). The Mann-Whitney test was used to assess the associations between the independent variables and the OHIP-14. Poisson regression models were also used in the analyses (α=0.05).

**RESULTS:**

In the PHC, of the 270 individuals invited to participate in the study, 164 (60.7%) were interviewed and clinically examined; while in the SC, of the 166 individuals invited to participate in the study, 124 (74.7%) were interviewed and clinically examined. After adjustments for sociodemographic, psychosocial and clinical factors, we found that the impact on OHRQoL was 2.8 times higher (95%CI 2.0–4.2) for older adults who did not participate in SC.

**CONCLUSION:**

Older adults who participated in SC showed better perception on OHRQoL, independently of sociodemographic, psychosocial and clinical factors.

## INTRODUCTION

The concept of quality of life is directly related to one’s degree of satisfaction with their family, social life, and health over the years^1^. Oral health is essential to individual general health and quality of life^2 , 3^. The American Dental Association defines oral health as a functional, structural, aesthetic, physiologic and psychosocial state of well-being. With aging, a significant decline in oral health may be observed, making older adults more frail, dependent and disabled^4^. Since oral health is part of general health, poor oral health could affect functional, psychological and social aspects of daily living. Based on the conceptual rationale for subjective measures of a broader view of oral health, the oral health-related quality of life (OHRQoL) measures were developed to determine the extent to which oral conditions affect individual and social behavior.

Psychosocial factors such as depression, mobility limitations and disabilities that affect activities of daily living (ADL)^5^ have been pointed out as strong predictors of older adults’ quality of life^6^and OHRQoL^7^. ADL can be a positive influence for healthy and happy aging, resulting in different degrees of well-being. Self-help groups for older adults may preserve cognitive and physical conditions, resulting in greater individual levels of resilience and low likelihood of illness^8 , 9^.

Senior Centers (SC) are places where older adults perform physical and intellectual activities and establish relationships with other groups and community members. Thus, the participation in SC may contribute to the maintenance of intellectual and motor capacities and to a healthy aging. Studies have assessed OHRQoL in older adults^10 , 11^, but the influence of their participation in SC on OHRQoL has not been evaluated.

Due to the aging process observed worldwide, such a knowledge is relevant to understand whether and to what extent SC could improve OHRQoL perception and, based on that, improve the formulation of health policies. Therefore, this study aimed to compare the OHRQoL perception in two independent samples: older adults who participate in SC and older adults who visited Primary Healthcare Centers (PHC) and do not participate in SC. Our hypothesis was that the participation in SC could be associated with improved OHRQoL perception.

## METHODS

### Study Design and Settings

This study was approved by the Local Human Ethics Committee (Protocol 913.653/2014) and is reported according to the observational study guide (STROBE)^12^. Written consent documents, based on the Declaration of Helsinki, were signed by all individuals who agreed to participate in the study.

A cross-sectional study was carried out from March 2014 to April 2016 in Pelotas, a Southern Brazilian city, with an estimated population of 343,651 inhabitants, of which 37,715 are older individuals, according to the 2010 Census, conducted by the Brazilian Institute of Geography and Statistics (IBGE)^13^. The study compared two independent samples of older adults: those who participate in SC and those who do not.

All participants of the *Centro de Extensão em Atenção à Terceira Idade da Universidade Católica de Pelotas* (CETRES—Extension Care Center for Older Adults of the Universidade Católica de Pelotas) were invited to participate in the study, which resulted in the partake of 124 older adults. The CETRES is a SC where people can engage in intellectual and/or physical activities aimed at healthy aging. This center has the greatest number of participants among the ones existing in the state (n = 166).

The group of older adults who do not participate in SC was composed of 164 individuals that attend to eleven PHC in the urban area of the city. The PHC was created by the Brazilian Ministry of Health to reorganize primary care. The sample was originated from a larger study, which started in 2009/2010 and included individuals aged 60 years or older, randomly selected from a list of 3,744 eligible older adults enrolled at 23 Family Health center provided by community health workers. A stratified simple randomization method was employed using a random number table. All 23 Family Health center in the city of Pelotas provided the name and sex of registered older individuals. A selection by lots was stratified by sex, based on the proportion of men and women enrolled at each of the PHC^11^. In the first follow-up, 270 older adults (61.6%) out of the 439 participants were found. Among these 270 individuals, 57 had died, 30 had moved to a different city and 19 declined to participate in this follow up. Thus, 164 people answered the questionnaire and underwent an oral health examination.

### Data Collection

Data collection consisted of interviewing the participants and performing an oral clinical examination on them. For both groups, all interviewers had been previously trained to administer the questionnaires. Dentists were trained and calibrated prior to clinical examination. The clinical examinations were done after the administration of the questionnaire, in order to guarantee blinding for the interview. Kappa statistic was used to assess inter-rater reliability.

### Interviews with Participants

Demographic and psychosocial information were collected. Demographics included sex (male and female); age at time of data collection (60–70 and ≥ 71); marital status (married or in a stable union, single, divorced, or widowed); and socioeconomic variables: educational level (> 8 years and ≤ 8 years of study) and household income (as categorized on the minimum wage in Brazil, ≤ 1.5 wages or more).

The psychosocial factors included measures to assess depression, happiness, sense of coherence and OHRQoL. The Hospital Anxiety and Depression Scale (HADS) was used to determine depression levels. HADS consists of 7 items to assess depression, with 4 answer choices in each item^5^ that correspond to a score ranging from 0, for the absence of symptoms, to 3, the maximum symptomatology^14^.

Andrews’ Question Scale was used to evaluate the participants’ level of happiness, ranging from high (A) to low (G); individuals were considered “happy” if they selected faces A or B^15^.

The Sense of Coherence Scale used in this study consists of 29 items scored with 7-point scales assessing three components: comprehensibility, manageability and meaningfulness. The higher scores indicate stronger sense of coherence^16^.

The Brazilian version of the Oral Health Impact Profile short-form (OHIP-14) assessed the OHRQoL outcome in this study. OHIP-14 is based on 7 domains, with two questions each scored from 0 to 4 points. Higher scores indicated worse OHRQoL^17^.

### Clinical Oral Examination

To obtain clinical information on oral health — use and need for any type of prosthesis and the index of decayed (D), missing (M), or filled (F) teeth (DMFT) –, five trained professionals examined the participants. They were seated under natural light in the social centers, healthcare units or at home, and the examiners followed the criteria for epidemiological surveys proposed by the World Health Organization^18^.

In the SC, the Kappa values obtained by the examiners ranged from 0.80 to 0.96 for the use of dental prosthesis, from 0.66 to 0.76 for the need for prosthesis, and from 0.91 to 0.94 for dental caries. The Kappa values in HC, on the other hand, ranged from 0.76 to 0.92 for the use of dental prosthesis, from 0.67 to 0.75 for the need for prosthesis, and from 0.87 to 0.95 for dental caries.

### Data Analysis

The statistical software STATA 12.0 (Stata Corp, College Station, USA) was used for the analyses. The data distribution pattern was analyzed, and the non-parametric Mann-Whitney test was used to assess the associations between the independent variables and the OHIP-14 scores and to obtain the means of the OHIP-14 domains. The frequency of scores from 0 to 2 (never to occasionally) and from 3 to 4 (fairly often to very often) were categorized per item into dichotomous variables (never/hardly ever = without impact or occasionally/fairly often/often= with impact) for the analyses shown in Tables 2 and 3. Poisson regression multivariate was used for OHRQoL analysis. Three models were created for the OHRQoL outcome, to determine the association between participation in SC and OHIP-14 scores. The multivariate analysis of OHIP-14 scores was controlled by sex, age, marital status, educational level, and income in Model 1; by all these variables plus sense of coherence, happiness and depressive symptoms in Model 2; and by all these variables plus number of teeth, use and need for dental prosthesis in Model 3. All confounding variables with p ≤ 0.20 in the unadjusted analysis were entered into the model and maintained regardless of the respective p values. Effect measures and 95% confidence intervals were obtained. A level of significance of 5% was adopted.

## RESULTS

The sociodemographic, psychological, and clinical data of the population (SC and PHC) are shown in Table 1 . The SC sample was composed essentially of women (84.6%), aged from 60 to 70 years (60.5%), with an educational level > 8 years (60.4%), who were single, divorced, or widowed (50.8%), and had a household income > 1.5 minimum wages (57.9%). Most older adults had no depression symptoms (89.0%), reported happiness (62.0%), wore prosthesis (74.2%), had a need for prosthesis (64.52%), and had ≤ 12 teeth (52.5%). OHIP scores were associated with age (p = 0.046), sense of coherence (p < 0.001), depression (p < 0.001) and happiness (p = 0.051) for older adults participating in SC. The PHC sample was also composed essentially of women (73.7%), aged ≥ 71 years (63.5%), with an educational level > 8 years (91.4%), who were single, divorced, or widowed (55.0%), and had a household income > 1.5 minimum wages (58.3%). Most older adults who did not participate in SC had symptoms of depression (54.2%), reported happiness (62.6%), wore prosthesis (86.2%), had no need for prosthesis (54.3%), and had ≤ 12 teeth (87.8%). In PHC, statistically significant differences of OHIP scores were observed according to household income (p = 0.043) and depression (p = 0.006). The mean values and standard deviation (SD) of the OHIP-14 scores obtained from the PHC and SC samples were, respectively, 8.50 (10.52) and 6.20 (8.69), p < 0.001.

**Table 1:** Sample distribution and bivariate analysis of sociodemographic, psychosocial and oral clinical conditions related to OHIP-14 scores in Primary Healthcare Centers and Seniors Centers.

Variables	Primary Healthcare Centers (PHC)				Seniors Centers (SC)			

	N	Mean	SD	P-value PHC	N	Mean	SD	P-value SC
**Sociodemographic **								

**Sex**								
Female	121	8.3	10.9	0.252	105	6.3	8.4	0.087
Male	43	8.8	9.4		19	5.1	9.7	
**Age **								
60-70	60	9.4	11.3	0.607	75	7.4	9.1	**0.046**
> 71	104	8.1	10.1		49	4.2	7.5	
**Educational Level**								
>8 years	150	8.6	10.7	0.653	75	5.9	7.9	0.841
≤ 8 years	14	6.5	7.7		49	6.5	9.7	
**Marital Status**								
Married/Stable Union	73	8.2	9.3	0.964	61	6.6	9.1	0.673
Single/Divorced/Widowed	89	8.9	11.9		63	5.7	8.2	
**Household Income**								
≤ 1.5 minimum wage	67	10.0	10.9	**0.043**	45	6.67	9.62	0.293
>1.5 minimum wage	93	7.3	10.1		62	4.49	6.73	

**Psychosocial **								

**Sense of Coherence**								
High	67	6.3	6.7	0.232	61	3.2	4.7	**<0.001**
Low	62	10.9	13.5		63	9.0	10.5	
**Depression**								
Normal	64	6.6	7.7	**0.006**	98	5.4	7.3	**<0.001**
High/Low	76	13.5	14.7		12	15.1	13.0	
**Happiness**								
High	89	8.1	8.8	0.659	77	5.1	7.9	**0.051**
Low	53	9.2	12.9		47	7.9	9.6	

**Clinical **								

**Use for Prosthesis**								
No	22	9.4	10.9	0.511	32	4.0	6.6	0.073
Yes	138	8.2	10.4		92	6.94	9.20	
**Need for Prosthesis**								
No	87	7.2	9.0	0.261	44	5.7	10.0	0.126
Yes	73	9.8	11.9		80	6.46	7.92	
**Number of Teeth**								
0–12	145	8.8	10.9	0.275	65	7.7	10.1	0.057
> 12	19	6.2	7.5		59	4.4	6.4	

*Values different from 164 (Primary Healthcare Centers) and 124 (Seniors Centers) are due to missing responses; p < 0.05 indicates statistically significant differences; Mann Whitney test; Standard Deviation (SD).

**Figure 1:**
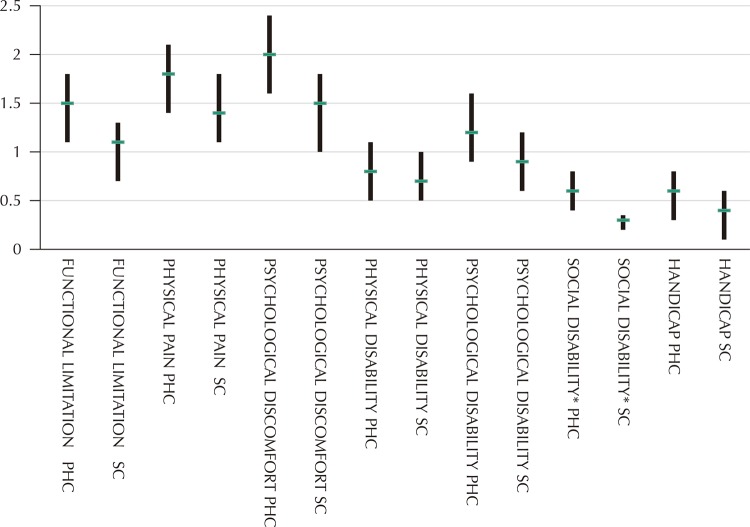
Comparison of OHIP-14 scores for all domains considering the Primary Healthcare Units (PHC) and Seniors Centers (SC).

Table 2 shows the mean values and SD of the OHIP-14 scores in the items of the seven assessed domains of those who participate and of those who do not participate in SC. Among older adults in PHC, the item “Worried due to problems with teeth, mouth or dentures” included in the Psychological discomfort domain showed the highest values (mean: 1.21, SD: 1.53), while the item “Has been unable to perform activities due to problems with teeth, mouth or dentures,” in the Handicap domain, had the lowest OHIP scores (mean: 0.16, SD: 0.56). When evaluating the older adults who participated in SC, the item “Has felt strong pain in the mouth,” included in the Physical pain domain, and the item “Has felt that life in general got worse due to problems with teeth, mouth or dentures,” of the Handicap domain, had the highest scores (mean: 0.56, SD: 0.23). The item “Has felt difficulty carrying out daily activities due to problems with teeth, mouth or dentures” of the Social Disability domain showed the lowest values (mean: 0.008, SD: 0.08). Overall, individuals who did not participate in SC had higher OHIP-14 scores and higher negative impact on OHRQoL than individuals who participate, with statistically significant differences in 7 of the 14 items evaluated by OHIP-14 scores. Figure 1 shows the OHIP-14 scores of SC participants and non-participants in these seven domains.

**Table 2:** Comparison of OHIP-14 scores for all domains considering the Primary Healthcare Centers and Seniors Centers.

OHIP-14 Domains	Primary Healthcare Centers			Seniors Centers			P-value

	Never/ Hardly ever	Often/ Ocasionally/ Very often	Mean (SD) OHIP-14	Never/ Hardly ever	Often/ Ocasionally/ Very often	Mean (SD) OHIP-14	
			
	n (%)	n (%)		n (%)	n (%)		
**FUNCTIONAL LIMITATION**							
1.Had difficulty to say a word due to problems with teeth, mouth or dentures	107 (73.30)	39 (26.7)	0.88 (1.39)	114 (91.94)	10 (8.06)	0.52 (1.09)	**0.018**
2.The taste of food has worsened due to problems with teeth, mouth or dentures	120 (83.33)	24 (16.67)	0.61 (1.22)	114 (91.94)	10 (8.06)	0.08 (0.27)	**< 0.001**
**PHYSICAL PAIN**							
3.Has felt strong pain in the mouth	116 (80.0)	29 (20.0)	0.68 (1.23)	117 (94.35)	7 (5.65)	0.56 (0.23)	0.285
4.Has felt uncomfortable eating some kind of food due to problems with teeth, mouth or dentures	97 (66.44)	49 (33.56)	1.06 (1.47)	104 (83.87)	20 (16.13)	0.16 (0.36)	**< 0.001**
**PSYCHOLOGICAL DISCOMFORT**							
5.Worried due to problems with teeth, mouth or dentures	89 (61.81)	55 (38.19)	1.21 (1.53)	103 (83.06)	21 (16.94)	0.16 (0.37)	**< 0.001**
6.Has felt stressed due to problems with teeth, mouth or dentures	111 (76.03)	35 (23.97)	0.79 (1.37)	108 (87.10)	16 (12.90)	0.12 (0.33)	**< 0.001**
**PHYSICAL DISABILITY**							
7.Was impaired to eat due to problems with teeth, mouth or dentures	126 (86.90)	19 (13.10)	0.42 (1.01)	120 (96.77)	4 (3.23)	0.32 (0.17)	0.277
8.Has stopped eating meals due to problems with teeth, mouth or dentures	127 (86.99)	19 (13.01)	0.42 (0.96)	120 (96.77)	4 (3.23)	0.32 (0.17)	0.253
**PSYCHOLOGICAL DISABILITY**							
9.Has had problems relaxing due to problems with teeth, mouth or dentures	129 (88.36)	17 (11.64)	0.42 (1.05)	121 (97.58)	3 (2.42)	0.24 (0.15)	0.060
10.Has felt ashamed due to problems with teeth, mouth or dentures	111 (76.55)	34 (23.45)	0.79 (1.34)	108 (87.10)	16 (12.90)	0.12 (0.33)	**< 0.001**
**SOCIAL DISABILITY**							
11.Has had difficulties carrying out daily activities due to problems with teeth, mouth or dentures	128 (87.67)	18 (12.33)	0.40 (1.07)	123 (99.19)	1 (0.81)	0.008 (0.08)	**< 0.001**
12.Has been irritated with other people due to problems with teeth, mouth or dentures	138 (94.52)	8 (5.48)	0.20 (0.74)	121 (97.58)	3 (2.42)	0.24 (0.15)	0.555
**HANDICAP**							
13.Has felt that life in general got worse due to problems with teeth, mouth or dentures	130 (89.04)	16 (10.96)	0.43 (1.06)	117 (94.35)	7 (5.65)	0.56 (0.23)	0.182
14.Has been unable to perform activities due to problems with teeth, mouth or dentures	140 (95.89)	6 (4.11)	0.17 (0.56)	122 (98.39)	2 (1.61)	0.16 (0.12)	0.846

* Mann-Whitney test with statistically relevant differences

The unadjusted regression model showed that individuals who did not participate in SC presented higher impact on OHRQoL. After the adjusted analysis for sociodemographic factors (model 1), the impact on OHRQoL was 2.8 times higher for older adults who did not participate in SC (PR: 2.8, 95%CI: 1.9–4.0). When the model was adjusted for sociodemographic (model 1) and psychosocial (model 2) factors, the impact was even higher (PR: 2.9, 95%CI: 2.0–4.3). Finally, after adjusting for sociodemographic, psychosocial, and clinical factors (model 3), the impact on OHRQoL was 2.8 times higher for older adults who did not participate in SC (PR: 2.8, 95%CI: 2.0–4.2) ( Table 3 ).

**Table 3:** Unadjusted and adjusted analyses of the Poisson Regression of OHIP-14 scores for the participation or not in Seniors Centers (SC).

Participation in SC	Unadjusted	Model 1	Model 2	Model 3
		
		Adjusted^**^	Adjusted^**^	Adjusted^**^
			
	RR (95%CI)	RR (95%CI)	RR (95%CI)	RR (95%CI)
	p < 0.001	p < 0.001	p < 0.001	p < 0.001
Yes	1.0	1.0	1.0	1.0
No	2.4 (1.84–3.13)	2.8 (1.9–4.0)	2.9 (2.0–4.3)	2.8 (2.0–4.2)

* Model 1: sex, age, marital status, educational level, family income.

** Model 2: model 1 variables plus sense of coherence, happiness and depressive symptoms.

*** Model 3: model 1 and 2 variables plus number of teeth, use and need for dental prosthesis.

95%CI: 95% Confidence Interval.

p < 0.05 indicates statistically significant differences; Mann Whitney test; Standard Deviation (SD).

## DISCUSSION

The aging process leads to changes in general health and, consequently, in oral health^19^. This study evaluated if the participation in SC influences the perception of OHRQoL. Our hypothesis was confirmed, since older adults who participated in SC had better perceptions of OHRQoL than those who did not.

In general, all independent variables evaluated in each group (PHC and SC) showed lower OHIP-14 scores for SC participants. When both groups were compared, statistically significant differences in the scores were observed for independent variables. The socioeconomic status has been considered an important predictor of oral health status, and consequently of OHRQoL^20 , 21^. Nevertheless, findings of this study showed that those who had the worst OHRQoL perception were the older adults who did not participate in SC, regardless of their household income.

Concerning psychosocial factors, depression was the only one associated with OHRQoL in both groups. Indeed, higher OHIP-14 scores were found for participants in SC. A possible explanation for this finding is that older adults seek SC to improve their daily lives and interact socially, especially with individuals of the same age. This behavior enables them to build new bonds of relationship and favors their physical, psychological and social well-being^22^. This is especially important because depression or depressive symptoms are the most common co-morbidity among older adults^23^, affecting their quality of life^24^ and OHRQoL^7^.

SC participants showed lower impacts (lower mean scores) on OHRQoL than those who did not participate in SC in most of the OHIP-14 domains, with statistically significant differences in 7 of the 14 evaluated items. The lowest impact occured in the social disability domain, in the question about daily activities. Probably, the better interaction and the relationships among SC participants result in social support for them. This understanding is in line with a previous study that reported social support has been hypothesized to contribute to better cognitive and physical health, results of some of its benefits such as increased social integration^24^. Studies have also shown that social support is linked to the presence of more functional teeth^25^ and better OHRQoL^26^.

The results of this study are substantial because even after the adjustment for sociodemographic, psychosocial and clinical factors, the regression analysis found that older adults who did not participate in SC had 2.8 times higher impact on OHRQoL.

These findings are important due to the large number of older adults worldwide. The aging-related chronic diseases require attention due to the aspects of increased life expectancy that accompany the aging process^1 , 2^. The factors that predispose people to impaired well-being and OHRQoL can change over time^24 , 25^. Because of this, even older adults with no functional limitation could present adverse effects over time due to the aging process. Thus, the participation in SC should be stimulated in Brazil in order to promote a heathy aging process for the population. However, although studies have been conducted in SC to evaluate the well-being of the participants^27^, this is the first epidemiological study evaluating the influence of the participation in SC on OHRQoL. This study has limitations. The individuals included in it were mostly women. Nonetheless, there was no association between the evaluated variables and the sex of the individuals in any of the groups. This finding only indicates the current situation of older adults in Brazil, a group in which women outnumber men^13^, seen women are more likely to adopt preventive health behaviors than men^28 , 29^. Furthermore, the cross-sectional design of this study was a limitation, since it precluded inferences about causal directions, especially because a negative or positive OHRQoL perception may not be a stable characteristic of an individual throughout life, being susceptible to environmental factors^22^. Our study also presents strengths that should be highlighted. The adequate calibration among examiners and the use of standardized and validated questionnaires assured its internal validity. The OHIP-14 is a widely used assessment tool for the measuring of negative impacts of oral problems on the lives of individuals. In addition, the use of the World Health Organization criteria allows comparisons with studies in the literature.

SC are important because a reduced social network may contribute to exacerbate disabilities or impose lifestyle limitations, leading to social isolation, which, in turn, may interfere with the health behaviors of older adults^9 , 30^. In a context in which little is known about the role of SC in the individual perception on OHRQoL, our findings contribute as an initial step towards the understanding of this relationship. Having in sight that the older adults will be the majority of the population in a few years, the incentive to participate in SC may be an important strategy and alternative to be adopted by policy makers for the improvement in the well-being of this age group, since SC seems to be closely related with individuals’ perceptions of OHRQoL. Therefore, this subject is relevant to public health and the findings of this study are likely to be useful for the planning of health services. Further studies evaluating the influences of older adults’ active participation in social activities in their communities should be conducted, since the adoption of physical and intellectual activities may enhance their well-being and reduce the loneliness dissatisfaction among them, promoting a health strategy throughout the life-course^8^.

Finally, the results of this study indicate that SC participants show better OHRQoL perception, independently of sociodemographic, psychosocial, and clinical factors. Thus, exploring interventions that increase the participation of older adults in SC could improve their well-being and promote healthy aging.
